# Altered functional connectivity of nucleus accumbens in patients with persistent postural-perceptual dizziness: a resting-state functional magnetic resonance imaging study

**DOI:** 10.3389/fpsyt.2026.1825054

**Published:** 2026-08-25

**Authors:** Dan Liu, Shuaishuai Shen, Guanyu Shi, Wenli Zhou, Yang Sun, Yueji Liu, Lijie Xiao, Liangqun Rong

**Affiliations:** 1 Second Affiliated Hospital, Xuzhou Medical University, Xuzhou, China; 2 Graduate School of Xuzhou Medical University, Xuzhou, China

**Keywords:** functional connectivity, persistent postural perceptual dizziness, resting state functional magnetic resonance imaging, superior occipital gyrus, the nucleus accumbens

## Abstract

**Objective:**

This study aimed to investigate alterations in functional connectivity (FC) of the nucleus accumbens (NAc) in patients with persistent postural-perceptual dizziness (PPPD) using resting-state functional magnetic resonance imaging (rs-fMRI), and to examine the relationship between aberrant FC and clinical characteristics.

**Methods:**

Twenty-four patients with PPPD and 25 age and gender matched healthy controls (HCs) were enrolled. Rs-fMRI data was preprocessed using DPABI. The bilateral NAc were selected as seed regions, and seed-to-voxel FC analysis was performed. FC differences between groups were assessed using two-sample t-tests with age, sex, HAMA, and HAMD scores as covariates (voxel-level FDR-corrected *q* < 0.05). Correlation analysis was conducted between abnormal FC values and clinical variables.

**Results:**

Compared with HCs, patients with PPPD exhibited significantly enhanced FC between the right NAc and the left superior occipital gyrus (Occipital_Sup_L; peak MNI: -24, -96, 24; T value = 4.35; voxel-level FDR-corrected *q* < 0.001), as well as between the right NAc and the right Inferior Frontal Gyrus, Orbital part (Frontal_Inf_Orb_R; peak MNI: 39, 45, -3; T value = 4.90; voxel-level FDR-corrected *q* < 0.001). No significant FC abnormalities were detected for the left NAc seed. Correlation analysis revealed a significant negative correlation between the FC value of right NAc to left superior occipital gyrus and disease duration in PPPD patients (*r* = -0.61, FDR-corrected *q* = 0.03).

**Conclusion:**

Patients with PPPD exhibit enhanced FC between the right NAc and visual- and emotion-related cortical regions, including the superior occipital gyrus and orbitofrontal cortex. The negative correlation with disease duration suggests that these FC alterations may be most pronounced in the early stages of the disorder. These findings provide new insights into the neural mechanisms of visual dependence and emotional dysregulation in PPPD.

## Introduction

Persistent postural perceptual dizziness (PPPD) is the most common chronic functional neurological disorder, with symptoms persisting for at least three months. It manifests as persistent non-rotational vertigo, dizziness, unsteadiness, or swaying sensations that occur on most days, severely affecting patients’ daily lives (1, 2). PPPD patients experience worsening dizziness in upright positions, during active or passive movement, and when exposed to complex or moving visual environments (3–5). The etiology and pathogenesis of PPPD remain incompletely understood. Current evidence suggests that PPPD may arise from dysfunction in the balance system and/or psychological factors, representing a chronic vestibular syndrome with complex interactions between sensory, motor, and emotional processing systems (6–11).

Previous neuroimaging studies have identified widespread abnormalities in brain regions involved in emotional processing, balance regulation, and visual-vestibular integration in PPPD patients. *Lee* et al. (12) demonstrated that patients with PPPD exhibited altered functional connectivity (FC) involving the default mode network, visual network, and cerebellum, with significantly decreased FC between the right nucleus accumbens (NAc) and the anterior left temporal fusiform cortex, providing evidence of NAc involvement in PPPD via resting-state functional MRI (rs-fMRI). *Riccelli* et al. (13) reported that PPPD patients showed altered insular and occipital responses to simulated vertical self-motion, indicating aberrant visual-vestibular processing at the cortical level. *Yagi* et al. (14) subsequently found changes in FC among vestibulo-visuo-somatosensory and spatial cognitive cortical areas following visual stimulation in PPPD patients, supporting the presence of multisensory integration dysfunction. More recently, *Li* et al. (15) employed graph theoretical analysis to identify abnormal neural circuits in PPPD, revealing disrupted topological properties in brain networks with key nodes involving the visual cortex, sensorimotor cortex, and subcortical regions. Collectively, these findings suggest that PPPD involves impaired integration of visual, vestibular, and somatosensory information (7, 16), which may underlie patients’ persistent dizziness and postural instability.

Despite these advances, specific neuroimaging biomarkers for PPPD have yet to be identified, and targeted treatment options remain limited. PPPD is a complex brain functional disorder that impairs not only balance control (4, 5, 16) but also significantly affects emotional regulation and cognitive function (6, 8, 17). Given the high comorbidity of anxiety and depressive symptoms in PPPD, understanding the neural mechanisms that link emotion-regulating circuits to vestibular-visual processing systems is of critical importance.

The striatum, particularly the NAc, is a key subcortical structure involved in reward processing, emotional regulation, and motor control (18–20). The NAc is located between the rostral basal forebrain and the preoptic area of the hypothalamus and is anatomically divided into core and shell subregions (18, 21). The shell primarily receives limbic information and is involved in emotional processing and learning, whereas the core receives motor-related inputs and participates in behavioral regulation (19, 20, 22). Importantly, resting-state FC studies have demonstrated that the human striatum, including the NAc, exhibits prominent functional connectivity with the orbitofrontal cortex (OFC), anterior cingulate cortex, and temporal-occipital regions, forming distinct corticostriatal circuits (23). These connections, particularly the dense bidirectional projections between the NAc and OFC via the mesolimbic dopamine pathway (24, 25), provide the structural foundation for the NAc’s role in integrating emotional, cognitive, and sensory information.

Although *Lee* et al. (12) identified NAc involvement in PPPD, whether the NAc exhibits aberrant FC with brain regions specifically involved in visual processing and emotional regulation, and how such alterations relate to clinical symptoms, remain unknown. The occipital cortex is the primary cortical hub for visual processing, and PPPD patients exhibit heightened visual dependence with occipital hyperactivation (13, 14). The OFC is critically involved in emotional appraisal and may contribute to the anxiety and fear responses commonly observed in PPPD (26, 27). However, no study has specifically examined NAc FC with these regions in PPPD using a seed-based approach. Therefore, the present study employed rs-fMRI to investigate FC changes of the bilateral NAc in PPPD patients and explored the correlations between aberrant FC and clinical characteristics. Given the well-documented visual-vestibular integration deficits and emotional dysregulation in PPPD, we hypothesized that PPPD patients would exhibit abnormal NAc FC specifically with visual and emotion-related cortical regions.

## Materials and methods

### Participants

This study enrolled PPPD patients from the Second Affiliated Hospital of Xuzhou Medical University between September 2022 and October 2024. Age and gender matched healthy controls (HC) were recruited from the hospital’s health examination center during the same period. This study complied with the Declaration of Helsinki and was approved by the Ethics Committee of the Second Affiliated Hospital of Xuzhou Medical University (approval number: 2022010305). All participants provided written informed consent prior to enrollment.

Inclusion criteria for PPPD patients were as follows: (1) meeting the diagnostic criteria for PPPD established by the Bárány Society in 2017 (3). First, for at least three months on most days, patients experienced one or more of the following symptoms-dizziness, postural instability, or non-rotational vertigo, with each episode typically lasting several hours and symptom severity fluctuating over time (symptoms were not required to be present throughout the entire day); Second, while persistent symptoms lacked specific triggers, they could be exacerbated by upright posture, active or passive movement in any direction or position, and exposure to moving visual stimuli or complex visual environments; Third, the disorder could be precipitated by acute, episodic, or chronic vestibular syndromes, as well as other neurological diseases or psychological conditions-when the triggering condition was acute or episodic, the initial symptoms conformed to the description in and after the triggering condition resolved, symptoms initially appeared intermittently and eventually progressed to a continuous course; when the triggering condition was a chronic syndrome, symptoms developed slowly and gradually worsened; Fourth, the symptoms caused clinically significant distress or functional impairment; Fifth, the symptoms were not better accounted for by another disease or disorder. (2) Right-handed individuals aged 18–70 years. (3) No history of drug or alcohol abuse. (4) Able to provide informed consent. Exclusion criteria for PPPD patients were: (1) contraindications for MRI examination; (2) evidence of massive cerebral infarction or hemorrhage on MRI; (3) severe white matter hyperintensities (Fazekas scale ≥ 3).

Inclusion criteria for HC were: (1) Right-handed, matched to the PPPD group for age and sex; (2) No history of vertigo or family history of vestibular disorders; (3) No history of mental illness; (4) No history of drug or alcohol abuse; (5) Voluntary participation with signed informed consent. The sole exclusion criterion for HC was contraindications for MRI examination.

### Clinical assessments

Demographic and clinical data collected for PPPD patients included age, gender, disease duration, and history of hypertension and diabetes. All patients completed the following standardized assessments: the Dizziness Handicap Inventory (DHI), a 25-item self-report questionnaire assessing the impact of dizziness on daily life across three subscales physical (DHI-P, 7 items, score range 0-28), emotional (DHI-E, 9 items, range 0-36), and functional (DHI-F, 9 items, range 0-36), with higher scores indicating greater handicap; the Hamilton Anxiety Rating Scale (HAMA), a 14-item clinician-administered scale measuring anxiety severity (each item rated 0-4, total range 0-56); and the Hamilton Depression Rating Scale (HAMD), a 17-item clinician-administered scale assessing depression severity (total range 0-52). HC provided demographic information (age, gender) and medical history (hypertension, diabetes) and completed both the HAMA and HAMD scales.

### Acquisition of MRI data

The magnetic resonance instrument in this study was performed using a GE Discovery MR750w 3.0T superconducting magnetic resonance imaging (MRI) system at the Second Affiliated Hospital of Xuzhou Medical University. All subjects’ MRI data were acquired from the same magnetic resonance equipment. During the scanning process, the subjects were asked to close their eyes, keep a resting state, keep calm and relaxed, and avoid falling asleep during the scanning process. Firstly, routine head MRI scan was performed to confirm the absence of organic brain lesions. Subsequently, blood oxygen levels dependent (BOLD) images were collected using an echo planar imaging (EPI) sequence. The specific parameters were as follows: repetition time (TR)=2000 ms, echo time (TE) =30 ms, flip angle (FA) = 90°, field of view (FOV) = 200 mm×200 mm, number of slices = 38, Matrix of acquisition=64×64, voxel size = 3.0 mm×3.0 mm×3.0 mm, slice thickness = 3 mm, interval = 0 mm, a total of 210 time points were obtained. T1-weighted anatomical images were acquired using 3D-BRAVO sequence, TR = 2500 ms, TE = 3.5 ms, FA = 8°, Matrix of acquisition = 256×256, slice thickness =1 mm, Number of slice = 156.

### fMRI data preprocessing

Functional image data were processed and analyzed using DPABI software (DPABI_V7.0_230110, http://rfmri.org/dpabi) running on MATLAB 2023a (MathWorks, Inc., Natick, MA, USA). The preprocessing steps were as follows: (1) Data format conversion: DICOM images acquired from the MRI scanner were converted to NIfTI format. (2) Removal of initial time points: The study acquired a single resting-state fMRI run of 210 volumes per participant (TR = 2000 ms, total duration = 7 minutes). The first 10 time points were discarded to minimize the influence of initial MRI signal instability and allow for T1-equilibration effects. (3) Slice timing correction: All 38 slices per volume were corrected for temporal offset relative to the first slice, which was selected as the reference slice. (4) Head motion correction: Realignment of all volumes to the first image was performed to correct for subtle head movements during scanning. Subjects with head motion exceeding 3 mm of maximum translation or 3°of maximum rotation in any direction were excluded. One HC subject was excluded due to excessive head motion. The mean framewise displacement (FD) was calculated for each subject. No significant difference was observed between groups [PPPD: mean FD = 0.06 (0.05,0.08) mm; HC: mean FD = 0.06 (0.04,0.09) mm; *P* = 0.83], confirming that head motion did not differentially affect the two groups. (5) Nuisance covariate regression: White matter signal, cerebrospinal fluid signal, and the Friston 24 head motion parameters (6 rigid-body parameters, their temporal derivatives, and the corresponding quadratic terms) were regressed out from the BOLD time series. Global signal regression was not applied in the primary analysis to avoid introducing spurious negative correlations. (6) Spatial normalization: The Diffeomorphic Anatomical Registration Through Exponentiated Lie Algebra (DARTEL) method was used to normalize functional images to the Montreal Neurological Institute (MNI) space, with resampling to a voxel size of 3 mm × 3 mm × 3 mm. (7) Band-pass filtering: A temporal band-pass filter (0.01–0.08 Hz) was applied to reduce the effects of low-frequency drift and high-frequency physiological noise. (8) Spatial smoothing: Images were smoothed with a Gaussian kernel of 6 mm × 6 mm × 6 mm full width at half maximum (FWHM) to improve the signal-to-noise ratio and to facilitate group-level analysis. No scrubbing (motion censoring) was applied, as no subjects exhibited FD spikes exceeding 0.5 mm in more than 10% of volumes.

### Seed-based functional connectivity analysis

**Figure 1:**
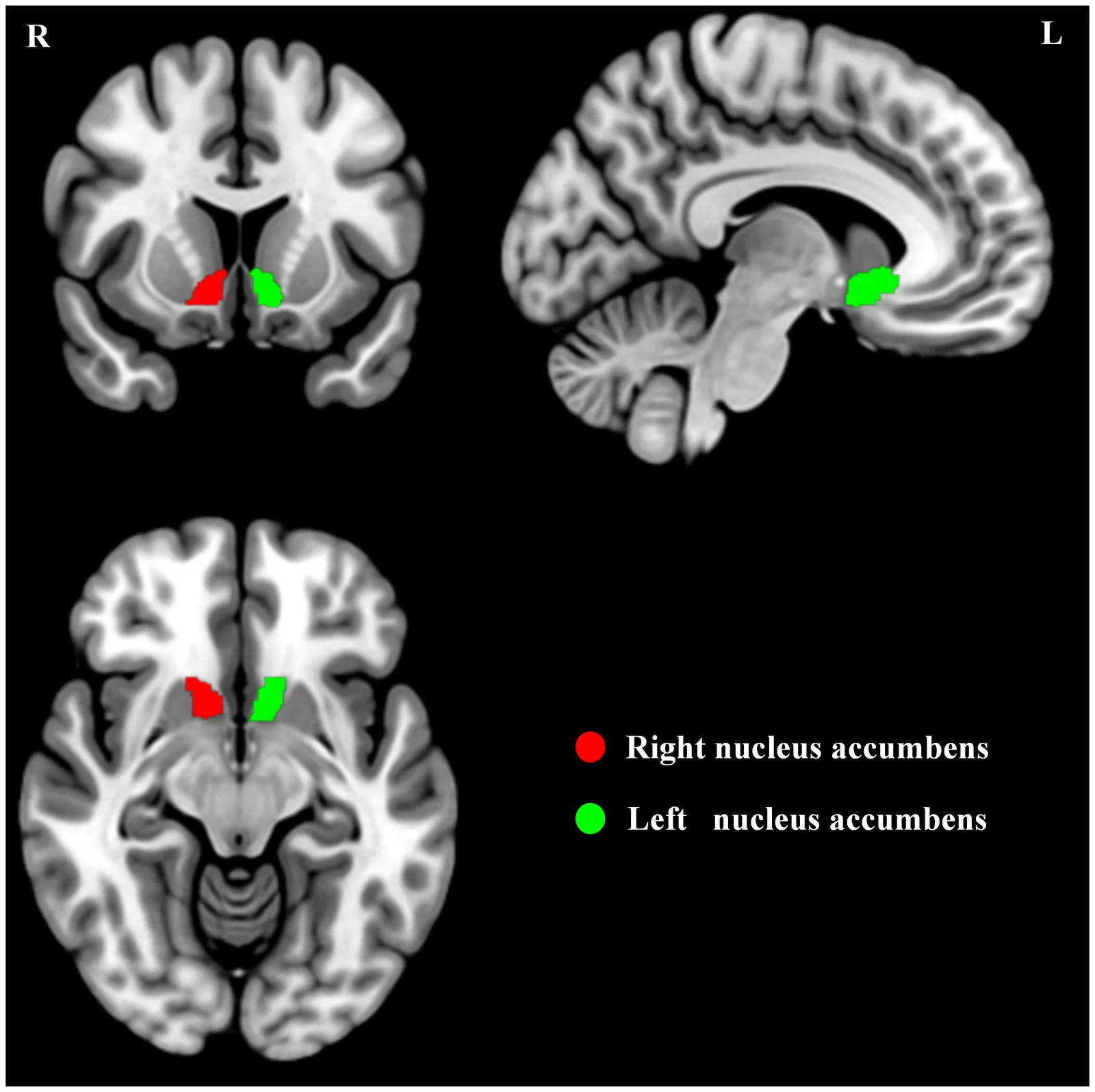
Bilateral nucleus accumbens seed regions. The NAc seed masks were defined using the AAL3 atlas. Red: right NAc; Green: left NAc. Masks are overlaid on the MNI152 standard brain template.

The bilateral NAc were selected as seed regions for FC analysis. The NAc seed masks were defined using the Automated Anatomical Labeling (AAL3) atlas (see **Figure 1**). For each seed, the mean BOLD time series was extracted from all voxels within the seed mask and converted to Fisher’s Z-scores to improve normality. Pearson correlation coefficients were then computed between the mean seed time series and the time series of all other voxels in the whole brain, generating individual voxel-wise FC maps for each subject.

To account for potential interhemispheric correlation between the bilateral NAc time series, the time series of the contralateral NAc was included as an additional nuisance regressor when analyzing the ipsilateral seed.

### Correlation analysis

To examine the relationship between neuroimaging alterations and clinical characteristics, we extracted the mean FC values from significant clusters identified in the group comparison. Spearman partial correlation analyses were then performed between these FC values and clinical variables (DHI total score, DHI subscale scores, and disease duration), controlling for age and gender, HAMA, HAMD as covariates. Multiple comparison correction was performed using the false discovery rate (FDR) method, with a significance threshold of *P* < 0.05.

### Statistical analysis

All clinical and demographic data were analyzed using R software (Version 4.5.3; R Foundation for Statistical Computing, Vienna, Austria). For continuous variables, normality was first assessed using the Shapiro-Wilk test. Normally distributed data were expressed as mean ± standard deviation (SD) and were compared between groups using independent-sample t-tests. Non-normally distributed data were expressed as median with interquartile range [M (Q25, Q75)] and were analyzed using the Mann-Whitney U test (rank-sum test). Categorical variables were expressed as frequencies and were compared using the χ² test or Fisher’s exact test. Statistical significance was set at *P* < 0.05 (two-tailed).

For rs-fMRI data, voxel-wise two-sample t-test was performed to compare FC between the PPPD and HC groups, with age, gender, HAMA scores, and HAMD scores included as covariates. Multiple comparison correction was performed using the false discovery rate (FDR) method at the voxel level, with a significance threshold of *q* < 0.05. As bilateral NAc seeds were analyzed separately, a Bonferroni correction was applied to account for the two seed comparisons, yielding an adjusted significance threshold of *q* < 0.025 (0.05/2). We note that both significant findings for the right NAc seed (*q* < 0.001, voxel-level FDR-corrected) easily survive this more stringent Bonferroni threshold. No significant FC alterations were detected for the left NAc seed even at the uncorrected threshold.

## Result

### Demographics and general clinical characteristics

**Figure 2:**
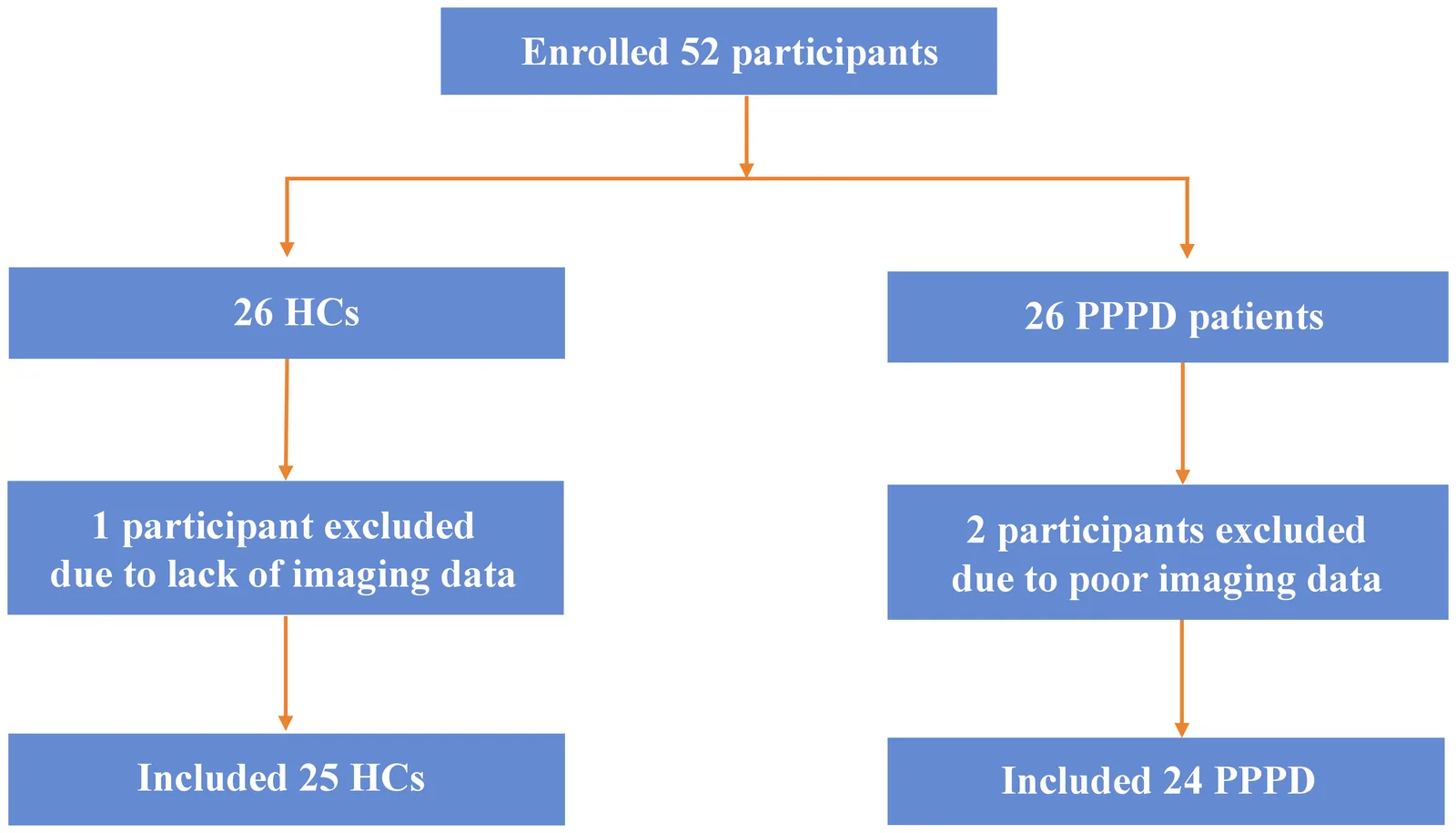
Flowchart of participant selection. A total of 26 patients with PPPD and 26 healthy controls were initially recruited. After exclusions (2 PPPD patients with missing imaging data; 1 HC with excessive head motion), the final sample comprised 24 patients with PPPD and 25 healthy controls.

This study initially enrolled 26 patients with PPPD, two of whom were excluded due to missing imaging data. A total of 26 HC were recruited, but one was excluded due to excessive head motion during MRI scanning. The final sample included 24 patients with PPPD and 25 HC (see **Figure 2**). There were no significant differences found between groups in age, gender, hypertension, or diabetes (*P* > 0.05, see **Table 1**). However, compared with HC, patients with PPPD had significantly higher HAMA and HAMD scores (*P* < 0.05, **Table 1**).

**Table 1:** Demographic and clinical characteristics of participants.

Variable	PPPD (*n* = 24)	HC (*n* = 25)	T/z/χ²	*P* value
Age (years)	59.00 (51.5, 62.5)	51.00 (41.25, 58.75)	1.66	0.10^c^
Gender (female/male)	13/11	10/15	0.50	0.48^b^
HAMD score	11.00 (8.75, 15.00)	4.00 (2.00, 6.00)	5.95	< 0.001^c^
HAMA score	14.75 ± 4.80	6.88 ± 1.33	7.74	< 0.001^a^
Diabetes (no/yes)	21/3	23/2	0.27	0.60^b^
Hypertension (no/yes)	20/4	22/3	0.22	0.64^b^
DHI score	60.00 (42.00, 68.50)	NA	NA	NA
DHI-P score	14.00 (10.00, 25.00)	NA	NA	NA
DHI-E score	19.58 ± 7.71	NA	NA	NA
DHI-F score	17.08 ± 8.94	NA	NA	NA
Duration (years)	0.67 (0.47, 1.25)	NA	NA	NA

^a^two sample t test; ^b^chi-square test; ^c^Mann-Whitney U test.

### NAc functional connectivity alterations between PPPD and HC.

**Figure 3:**
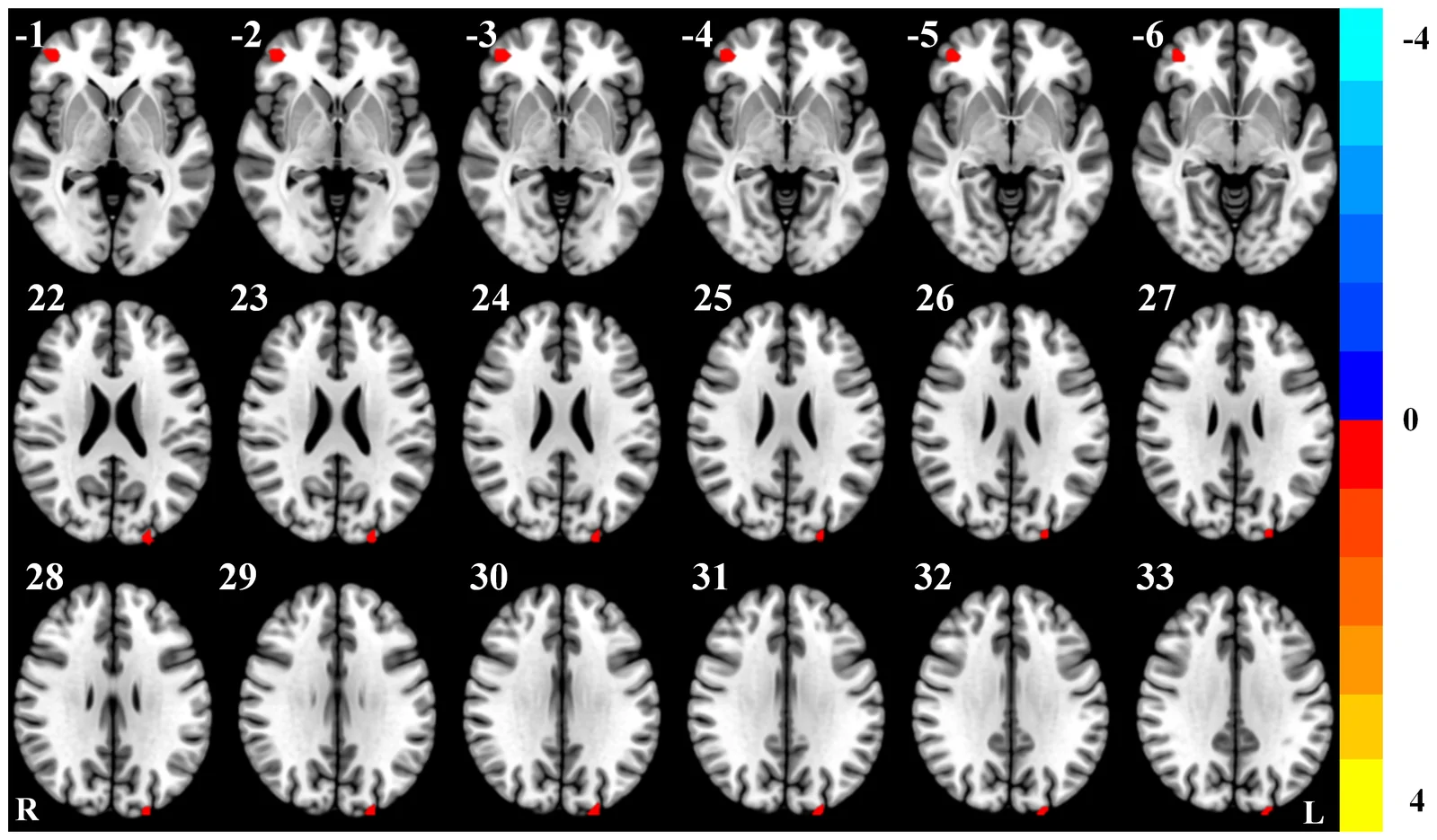
Brain regions showing significantly altered functional connectivity with the right NAc in PPPD patients compared with HC. Red clusters indicate significantly increased FC in the PPPD group (voxel-level FDR-corrected *q* < 0.001). Color bar represents T values.

**Figure 4:**
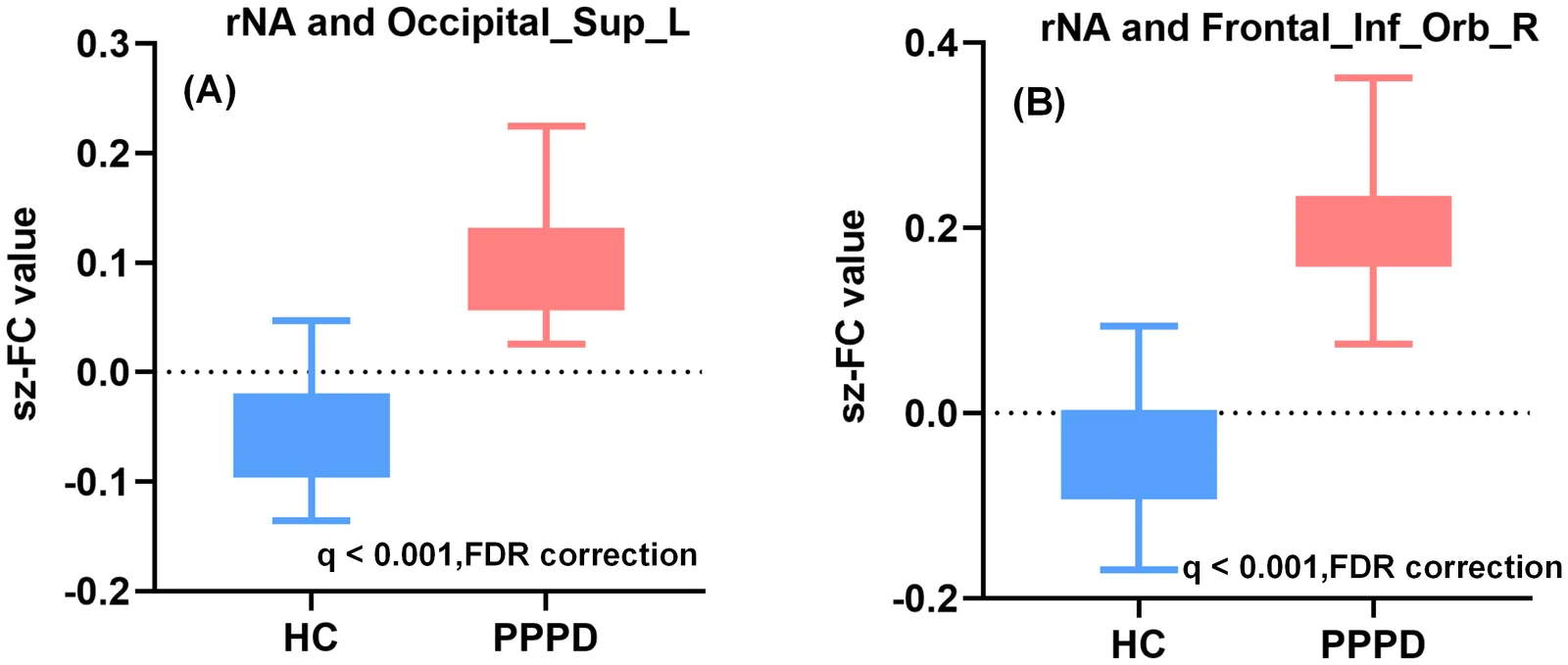
Comparison of z-FC values between groups PPPD and HC. **(A)** Right NAc-left superior occipital gyrus FC; **(B)** Right NAc-right OFC FC. Blue bars: healthy controls (*n* = 25); Red bars: PPPD patients (*n* = 24). Voxel-level FDR-corrected *q* < 0.001.

Seed-based FC analysis with the bilateral NAc as seeds revealed that, compared with HC, patients with PPPD exhibited significantly enhanced FC between the right NAc and the left superior occipital gyrus (Occipital_Sup_L; peak MNI: -24, -96, 24; T value = 4.35; cluster size = 30 voxels; voxel-level FDR-corrected *q* < 0.001, see **Table 2**; **Figures 3**, **4**), as well as between the right NAc and the right orbital part of the inferior frontal gyrus (Frontal_Inf_Orb_R, corresponding to the orbitofrontal cortex; peak MNI: 39, 45, -3; T value = 4.90; cluster size = 28 voxels; voxel-level FDR-corrected *q* < 0.001; see **Table 2**; **Figures 3**, **4**). No significant FC differences were detected when using the left NAc as the seed.

**Table 2:** Brain regions showing altered FC with the right NAc in PPPD patients compared with HCs.

Brain area	MNI (x, y, z)	Voxel size	T value	*P* value	η² value
Occipital_Sup_L	-24, -96, 24	30	4.35	< 0.001	0.31
Frontal_Inf_Orb_R	39, 45, -3	28	4.90	< 0.001	0.36

MNI: montreal neurological institute; R: right; L: left.

### Correlation analysis

**Figure 5:**
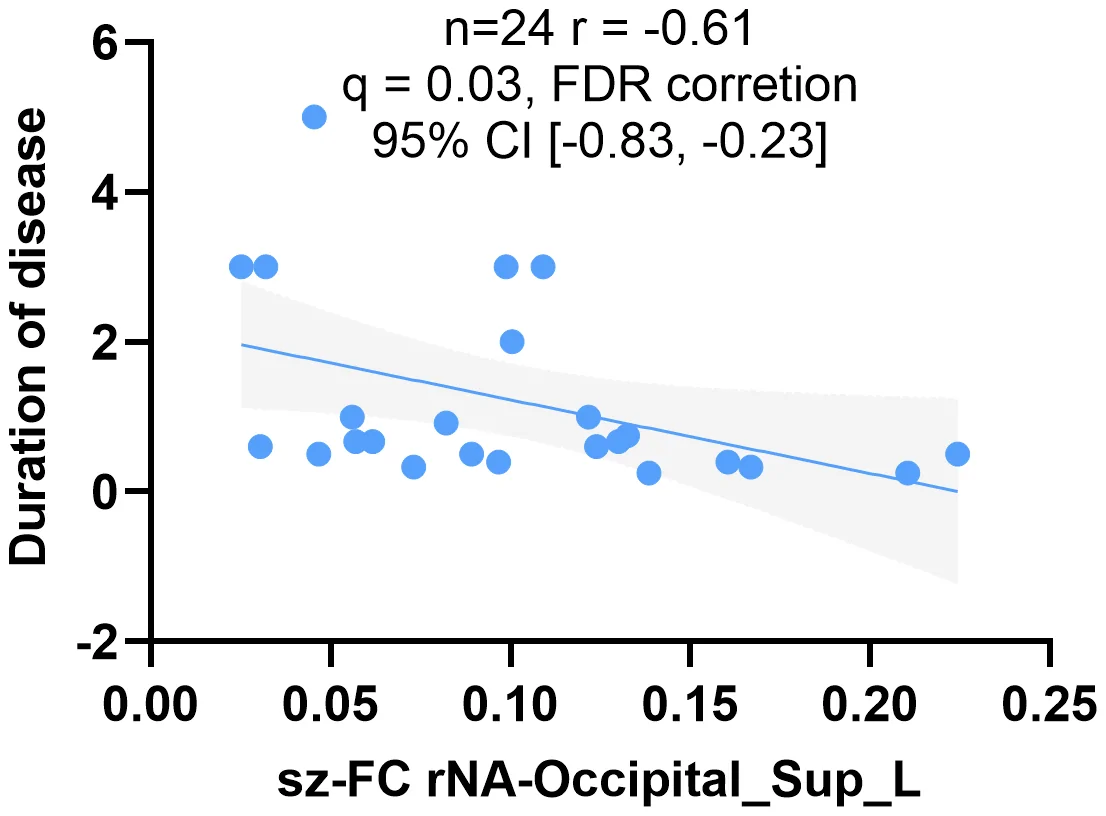
Correlation analysis between the sz-FC of right NAc to left superior occipital gyrus and disease duration in PPPD patients. Correlation analysis revealed a significant negative correlation between the FC values of the right NAc to left superior occipital gyrus and disease duration in patients with PPPD. [*r* = -0.61, *q* = 0.03, 95% CI (-0.83, -0.23), FDR-corrected].

Correlation analysis revealed a significant negative correlation between the FC values of the right NAc-left superior occipital gyrus and disease duration in patients with PPPD [*r* = -0.61, *q* = 0.03, 95% CI (-0.83, -0.23), FDR-corrected; see **Figure 5**]. No significant correlations were found between FC values and DHI, HAMA, HAMD scores after FDR correction (*P* > 0.05).

## Discussion

In this study, we used rs-fMRI to investigate FC abnormalities of the bilateral NAc in patients with PPPD and examined the relationship between aberrant FC and clinical characteristics. We found that compared with healthy controls (HC), PPPD patients exhibited significantly enhanced FC between the right NAc and the left superior occipital gyrus (Occipital_Sup_L), as well as between the right NAc and the right orbital part of the inferior frontal gyrus (Frontal_Inf_Orb_R), which corresponds to the lateral orbitofrontal cortex (OFC). No significant FC abnormalities were detected when using the left NAc as the seed. Furthermore, correlation analysis revealed that the FC between the right NAc and the left superior occipital gyrus was negatively correlated with disease duration in PPPD patients. These findings provide new evidence that NAc FC alterations in PPPD primarily involve visual and emotion-related cortical regions and may represent the neural substrates underlying the visual dependence and emotional dysregulation that characterize this disorder.

### The functional connectivity of rNAc to superior occipital gyrus and visual dependence in PPPD

The enhanced FC between the right NAc and the left superior occipital gyrus is consistent with the growing body of literature implicating the occipital cortex in PPPD pathophysiology. The occipital cortex is the primary cortical region for visual processing, and the superior occipital gyrus is specifically involved in higher-order visual functions, including visuospatial attention and motion perception (28). PPPD is fundamentally characterized by marked visual dependence, whereby patients experience exacerbated dizziness in visually complex or moving environments (2, 4, 5).

Several previous neuroimaging studies have demonstrated occipital cortex abnormalities in PPPD. *Riccelli* et al. (13) reported that during visual self-motion simulation, PPPD patients exhibited increased occipital and insular responses compared with HC, suggesting occipital hyperexcitability in response to visual stimuli. *Lee* et al. (12) found altered FC between the default mode network and occipital regions in PPPD, including the occipital fusiform gyrus and lateral occipital cortex. *Yagi* et al. (14) further demonstrated enhanced FC between visual and somatosensory areas in PPPD patients after visual stimulation, indicating that visual network dysfunction in PPPD extends to multisensory integration. Moreover, *Li* et al. (15) recently identified the occipital visual cortex as a key node in abnormal neural circuits in PPPD using network-based statistics, further emphasizing the central role of visual processing dysfunction in this condition. Our finding of enhanced NAc-occipital FC extends this literature by demonstrating that the NAc, a limbic-motor interface, shows aberrant coupling with the visual cortex in PPPD, suggesting that visual processing abnormalities may be influenced by subcortical emotional and motivational circuits.

We speculate that the enhanced NAc-superior occipital gyrus FC may reflect aberrant emotional salience attribution to visual information in PPPD. The NAc is a critical node in the brain’s salience and reward circuitry (20, 29). Enhanced coupling between the NAc and visual cortex could indicate that visual stimuli are abnormally “tagged” with emotional or motivational significance in PPPD patients, leading to heightened vigilance toward visual environments and exaggerated dizziness perception. This interpretation aligns with the clinical observation that PPPD patients frequently develop anticipatory anxiety and hypervigilance toward visual stimuli that trigger their symptoms (4, 10). The negative correlation between NAc-occipital FC and disease duration further supports this interpretation: enhanced FC may represent an early or active phase of the disorder when visual hypersensitivity is most pronounced, with gradual adaptive changes over longer disease courses. Neuroanatomically, the NAc receives indirect visual input via the amygdala and ventral temporal cortex (30, 31), providing a plausible polysynaptic pathway for the observed FC enhancement.

### The functional connectivity of rNAc-orbitofrontal cortex and emotional dysregulation in PPPD

The second major finding is the enhanced FC between the right NAc and the right OFC in PPPD patients. The OFC is a prefrontal region critically involved in emotional appraisal, reward evaluation, and affective regulation (26, 27). The OFC and NAc are anatomically and functionally connected via the mesolimbic dopamine pathway, forming a circuit that assigns emotional value to sensory stimuli and guides motivated behavior (23, 24).

The enhanced NAc-OFC FC in PPPD may represent a neural correlate of the emotional disturbances commonly associated with this condition. PPPD patients frequently present with comorbid anxiety, depression, and phobic avoidance behaviors (6, 8, 17). The NAc-OFC circuit is central to processing both rewarding and aversive signals, and dysfunction in this circuit has been implicated in anxiety disorders and major depression (27). Enhanced FC between these regions could reflect maladaptive emotional appraisal of dizziness-related sensory inputs, wherein negative affective responses are amplified through aberrant NAc-OFC communication. This interpretation is supported by the observation that our PPPD cohort had significantly higher HAMA and HAMD scores than HC, and we controlled for these emotional variables in our statistical model, suggesting that the NAc-OFC FC enhancement is at least partially independent of state anxiety or depression severity and may represent a more trait-like feature of PPPD-related emotional dysregulation.

The right-lateralization of our findings deserves comment. The right hemisphere is dominant for visuospatial attention, vestibular processing, and the regulation of negative affect (32, 33). Most previous PPPD studies have reported predominantly right-hemisphere abnormalities (12, 13, 16), and the right OFC has been implicated in the inhibition of behavioral responses to aversive stimuli (26). Our right-lateralized NAc-OFC findings are therefore consistent with the emerging consensus of right-hemisphere dysfunction in PPPD.

### Comparison with previous NAc findings in PPPD

Our results differ from those of *Lee* et al. (12), who reported decreased FC between the right NAc and the left anterior temporal fusiform cortex in PPPD using an independent component analysis (ICA) approach. Several methodological factors may account for this discrepancy. First, ICA analysis and seed-based FC analysis capture different aspects of functional organization; seed-based approaches are more sensitive to the specific connectivity profile of a predefined region. Second, our analysis incorporated comprehensive control for HAMA and HAMD scores-which was not performed in Lee’s study-and this control may have unmasked NAc connectivity patterns more specifically related to PPPD pathophysiology rather than to comorbid emotional symptoms. Third, differences in demographic and clinical characteristics between the two study samples may have contributed to divergent findings. Despite these differences, both studies converge on the important conclusion that the NAc is a functionally relevant node in the neural circuitry underlying PPPD, warranting further investigation.

### Correlation between FC alterations and clinical features

The negative correlation between right NAc-left superior occipital gyrus FC and disease duration in PPPD patients provides important temporal context for understanding the observed FC alterations. This finding suggests that enhanced NAc-occipital coupling may be most prominent in the early stages of PPPD, possibly reflecting an active phase of maladaptive visual-emotional integration. Over longer disease durations, the brain may engage compensatory mechanisms that gradually attenuate this aberrant coupling. Alternatively, patients with longer disease duration may have developed adaptive strategies (e.g., avoidance of triggering visual environments) that reduce the demand on this circuit. Future longitudinal studies tracking FC changes over the course of PPPD are needed to clarify the temporal dynamics of these alterations.

### Limitations

Several limitations of this study should be acknowledged. First, the cross-sectional design precludes causal inferences about whether the observed NAc FC alterations precede or result from PPPD; future longitudinal intervention studies are needed to address this question. Second, the sample size is relatively modest, and these findings should be considered exploratory and require replication in larger, independent cohorts. Third, this study is limited to seed-based FC analysis; future research should incorporate multidimensional approaches, including whole-brain network analysis and graph theory. Fourth, although we controlled HAMA and HAMD scores, residual confounding from other unmeasured psychological factors cannot be excluded. Fifth, the mean age of our cohort was approximately 52 years, an age range in which striatal dopamine signaling undergoes accelerated age-related changes (34); future studies across a broader age range are needed to examine potential age interactions with NAc FC alterations.

## Conclusion

This study demonstrates that patients with PPPD exhibit enhanced FC between the right NAc and the left superior occipital gyrus, as well as between the right NAc and the right orbitofrontal cortex. These findings suggest that NAc FC alterations in PPPD involve aberrant coupling with the visual cortex, which may contribute to the visual dependence that characterizes this disorder, and enhanced connectivity with the orbitofrontal cortex, which may reflect emotional dysregulation mechanisms. The negative correlation between NAc-occipital FC and disease duration further suggests that these alterations may be most prominent in the early stages of the disorder. Together, these results provide new insights into the neural mechanisms of PPPD and highlight the NAc as a potential target for future research on non-invasive neuromodulation.

## Data Availability

The study’s material, including raw data, statistical maps, ROI masks, or analysis scripts can be shared upon reasonable request.
